# The role of p53 in anti-tumor immunity and response to immunotherapy

**DOI:** 10.3389/fmolb.2023.1148389

**Published:** 2023-08-01

**Authors:** Lindsey Carlsen, Shengliang Zhang, Xiaobing Tian, Arielle De La Cruz, Andrew George, Taylor E. Arnoff, Wafik S. El-Deiry

**Affiliations:** ^1^ Laboratory of Translational Oncology and Experimental Cancer Therapeutics, Warren Alpert Medical School, Brown University, Providence, RI, United States; ^2^ Department of Pathology and Laboratory Medicine, Warren Alpert Medical School, Brown University, Providence, RI, United States; ^3^ Joint Program in Cancer Biology, Lifespan Health System and Brown University, Providence, RI, United States; ^4^ Legorreta Cancer Center, Brown University, Providence, RI, United States; ^5^ Pathobiology Graduate Program, Warren Alpert Medical School, Brown University, Providence, RI, United States; ^6^ Hematology-Oncology Division, Department of Medicine, Lifespan Health System and Warren Alpert Medical School, Brown University, Providence, RI, United States

**Keywords:** p53, immunotherapy, anti-cancer immunity, tumor microenvironment, DNA damage

## Abstract

p53 is a transcription factor that regulates the expression of genes involved in tumor suppression. p53 mutations mediate tumorigenesis and occur in approximately 50% of human cancers. p53 regulates hundreds of target genes that induce various cell fates including apoptosis, cell cycle arrest, and DNA damage repair. p53 also plays an important role in anti-tumor immunity by regulating TRAIL, DR5, TLRs, Fas, PKR, ULBP1/2, and CCL2; T-cell inhibitory ligand PD-L1; pro-inflammatory cytokines; immune cell activation state; and antigen presentation. Genetic alteration of p53 can contribute to immune evasion by influencing immune cell recruitment to the tumor, cytokine secretion in the TME, and inflammatory signaling pathways. In some contexts, p53 mutations increase neoantigen load which improves response to immune checkpoint inhibition. Therapeutic restoration of mutated p53 can restore anti-cancer immune cell infiltration and ameliorate pro-tumor signaling to induce tumor regression. Indeed, there is clinical evidence to suggest that restoring p53 can induce an anti-cancer immune response in immunologically cold tumors. Clinical trials investigating the combination of p53-restoring compounds or p53-based vaccines with immunotherapy have demonstrated anti-tumor immune activation and tumor regression with heterogeneity across cancer type. In this Review, we discuss the impact of wild-type and mutant p53 on the anti-tumor immune response, outline clinical progress as far as activating p53 to induce an immune response across a variety of cancer types, and highlight open questions limiting effective clinical translation.

## 1 Introduction

Approximately 50% of cancer patients have tumors with one or more genetic alterations in the tumor suppressor p53, making it the most frequently mutated gene in cancer. In response to DNA damage, hypoxia, oncogene activation, or ribosomal stress, p53 acts as a transcription factor to activate target genes that mediate various cell fates including apoptosis, cell cycle arrest, DNA damage repair, metabolic changes, and more (Hafner et al., 2019). p53 regulates hundreds of target genes which are carefully selected based on the desired cell fate. p53 is expressed in a wide variety of tissues and cell types, but variations in expression level and target gene selection result in a variety of cellular fates across different cell types (Burns et al., 2001; Fei et al., 2002). p53 activation may be accomplished by reactivating wild-type p53 function, for example, by inhibiting its negative regulator MDM2 (Shangary and Wang, 2008) or by restoring wild-type function to mutant p53, for example, by altering protein confirmation or inducing transcription of select target genes (Martinez, 2010). Numerous recent advancements as far as targeting p53 highlight its therapeutic potential (Zhang et al., 2022).

**FIGURE 1:**
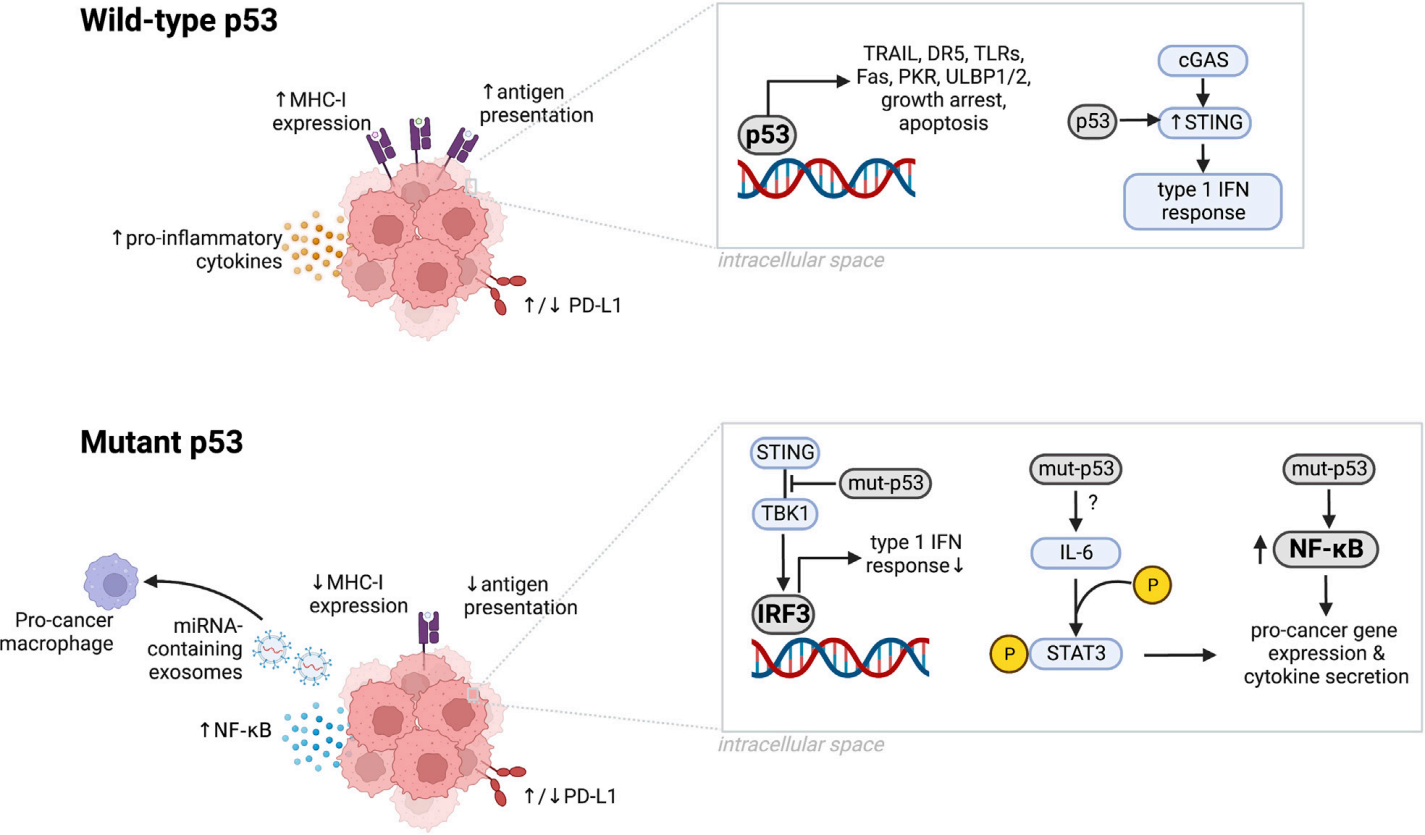
The microenvironment of tumors with wild-type or mutant p53. Wild-type p53 upregulates MHC-I expression, antigen presentation, secretion of proinflammatory cytokines, and immunostimulatory genes including TRAIL, DR5, TLRs, PKR, and ULBP1/2. Wild-type p53 also upregulates STING to support the cGAS/STING pathway, induces growth arrest, growth suppression, and apoptosis, and can upregulate or downregulate PD-L1 depending on the cancer type. p53 dysfunction induced by mutations can downregulate MHC-I expression, antigen presentation, and IRF3 activity while increasing NF-κB levels, NF-κB signaling and IL-6-mediated STAT3 phosphorylation. Mutant p53 can upregulate or downregulate PD-L1 depending on the cancer type. Created in BioRender.

p53 activation in cancer cells can impact the immune system through several mechanisms (Figure 1). p53 activation induces expression of immune-related genes such as tumor necrosis factor-related apoptosis-inducing ligand (TRAIL) (Kuribayashi et al., 2008; Cardoso Alves et al., 2021), death receptor 5 (DR5) (Wu et al., 1997), toll-like receptors (TLRs) (Muresan et al., 2020; Muresan et al., 2020), Fas (Müller et al., 1998), and ULBP1/2 (Textor et al., 2011) (Table 1); engages the cyclic GMP–AMP synthase (cGAS)–stimulator of IFN genes (STING) pathway (Ghosh et al., 2021; Pu et al., 2021; Ghosh et al., 2022); modulates levels of programmed death-ligand 1 (PD-L1) (Wang Y. et al., 2018; Thiem et al., 2019; Yadollahi et al., 2021); and promotes cytotoxic T cell-induced tumor cell death (Braun and Iwakuma, 2016). Experimental and clinical evidence suggests that mutated or non-functional p53 induces chronic inflammation in cancer cells and promotes an immunosuppressed tumor microenvironment (Cui and Guo, 2016; Agupitan et al., 2020), however it is also a tumor antigen (DeLeo et al., 1979) that can enhance response to immunotherapy (Dong et al., 2017; Biton et al., 2018; Deniger et al., 2018; Chasov et al., 2021). Together, these data suggest that p53 plays an important role in the modulation of anti-tumor immunity.

**TABLE 1:** p53-regulated genes that impact immune function.

p53-regulated gene	Up/down regulation by p53	Function	Ref.
CCL2	↑	Pro-cancer: can promote polarization of M2 macrophages	Hacke et al. (2010), Fei et al. (2021)
DR5	↑	Anti-cancer: also known as TRAIL-R2, receptor for TRAIL that promotes cellular apoptosis upon TRAIL binding	Wu et al. (1997)
Fas	↑	Anti-cancer: FasL receptor that promotes extrinsic cellular apoptosis pathways upon ligand binding	Müller et al. (1998)
IRF5	↑	Anti-cancer: activates the transcription of pro-inflammatory cytokines and mediates apoptosis in cancer cells	Mori et al. (2002), Barnes et al. (2003), Hu et al. (2005), Honda and Taniguchi (2006), Yanai et al. (2007), Couzinet et al. (2008), Hu and Barnes (2009)
IRF9	↑	Anti-cancer: contributes to p53-mediated upregulation of IFNs	Muñoz-Fontela et al. (2008)
		Pro-cancer: may promote cancer cell survival via IL6 & STAT3 activation	
PD-L1	↑/↓	Pro-cancer: binding to receptor PD-1 suppresses CD8^+^ T-cell activation	Cortez et al. (2016), Thiem et al. (2019), Costa et al. (2020)
PKR	↑	Anti-cancer: supports p53-mediated tumor suppression, mediates inflammasome activation and release of HMGB1	Yoon et al. (2009), Lu et al. (2012)
TLRs	↑	Anti-cancer: stimulates pro-inflammatory cytokines in cancer cells & lymphocytes	Muresan et al. (2020), Muresan et al. (2020)
TRAIL	↑	Anti-cancer: DR4/5 ligand expressed primarily by immune cells	Kuribayashi et al. (2008)
ULBP1/2	↑	Anti-cancer: enhances NK cell target recognition	Textor et al. (2011)

Evidence supporting the crucial role of p53 in the anti-tumor immune response has driven interest in the clinical translation of therapies involving p53 activation to induce an immune response with or without immunotherapy (Meric-Bernstam et al., 2017; Chung et al., 2019; Fang et al., 2019; Zhou et al., 2021). p53-restoring compounds and p53-based vaccines −/+ immune checkpoint inhibition (ICI) have been evaluated in clinical trials (Table 2). In this Review, we discuss the role of wild-type p53 in the anti-tumor immune response, the impact of p53 dysfunction, clinical progress as far as activating p53 to induce an immune response across a variety of cancer types, and open questions limiting effective clinical translation.

**TABLE 2:** Clinical trials involving p53 activation to induce an immune response.

ID	Intervention	Cancer Type	Status	Outcome	Ref.
*p53 activation + immune checkpoint inhibition*					
NCT04785196	APG-115 + toripalimab	Liposarcoma & advanced solid tumors	Recruiting	Not yet available	
NCT03611868	APG-115 + pembrolizumab	Solid tumors	Recruiting	Not yet available	Fang et al. (2019)
NCT04383938	PRIMA-1Met (APR-246) + pembrolizumab	Solid tumors	Completed	Acceptable safety profile; clinical activity in patients with solid tumors	Dumbrava et al. (2021), Park et al. (2022)
*p53 vaccines -/+ immune checkpoint inhibition*					
NCT01191684	p53MVA	Colorectal, stomach, or pancreatic cancer	Completed	Elevated p53-specific CD8+ T cell responses; clinical response not apparent	Chung et al. (2019)
NCT03113487	p53MVA + pembrolizumab	Recurrent ovarian, primary peritoneal, or fallopian tube cancer	Active, not recruiting	Not yet available	
NCT02432963	p53MVA + pembrolizumab	Solid tumors that have failed prior therapy	Active, not recruiting	3/11 patients SD for 30, 32, and 49 weeks with p53-specific T cells; 7/11 patients PD before 10 weeks, minimal p53-specific T cell responses, no clinical benefit	Chung et al. (2019)
NCT03406715	Ad.p53-DC + nivolumab & ipilimumab	Small cell lung cancer	Terminated	Not yet available	
NCT01639885	SLP-p53 + IFN-α2b	Platinum-resistant ovarian cancer	Completed	Not yet available	
NCT00844506	SLP-p53 + cyclophosphamide	Ovarian cancer	Completed	Not yet available	
NCT00617409	Paclitaxel -/+ INGN 225	Small cell lung cancer	Completed	Positive immune response in 20% of paclitaxel -/+ INGN 225 arm. Failed to improve ORR compared to paclitaxel alone.	Chiappori et al. (2019)
Unknown	ALVAC-p53	Advanced colorectal cancer	Completed	Potent T-cell and IgG responses induced in the majority of the patients	van der Burg et al. (2002)

SD, stable disease; PD, progressive disease

## 2 Wild-type p53 and the anti-tumor immune response

Reactivating wild-type p53 or restoring mutant p53 in cancer cells activates the innate immune system (Menendez et al., 2013), demonstrating clear involvement of p53 in the anti-tumor immune response. For example, the p53-reactivating compound APG-115 synergizes with ICI (Fang et al., 2019) and the efficacy of another p53-reactivating compound DS-5272 is dependent on natural killer (NK) cell activity (Hayashi et al., 2019). In this section, we discuss the role of wild-type p53 in the anti-cancer immune response.

### 2.1 p53 induces expression of immunomodulatory genes

p53 regulates the expression of hundreds of genes, many of which are involved in the immune response to cancer including TRAIL, DR5, TLRs, Fas, PKR, ULBP1/2, and CCL2.

TRAIL is a p53 target gene (Kuribayashi et al., 2008) that is primarily expressed by immune cells such as NK cells, T-cells, natural killer T (NKT) cells, dendritic cells (DCs) and macrophages (Smyth et al., 2003; Falschlehner et al., 2009). TRAIL binds to the p53 target gene DR5 (Wu et al., 1997) to induce apoptosis in a wide variety of cancer types while maintaining cancer cell specificity (Smyth et al., 2003), making it an attractive target for combination with immunotherapy. TRAIL compounds and agonists targeting DR5 (Dubuisson and Micheau, 2017) for treatment of numerous cancer types are highly developed in the clinic, providing further rationale to investigate this cytokine further as a widely applicable p53 target that may induce apoptosis and an immune response across numerous cancer types (Falschlehner et al., 2009; Ralff and El-Deiry, 2018). Further characterization of noncanonical TRAIL pathways and potential immunosuppressive effects of TRAIL treatment are needed prior to clinical translation (Sag et al., 2019; Cardoso Alves et al., 2021).

TLR3, 5, 7, 8, and 9 are primarily involved in the innate immune response and trigger type I interferon (IFN) synthesis through IFN regulatory factors (IRFs) (Sameer and Nissar, 2021). p53 can directly activate IRF5 (Mori et al., 2002) and IRF9 (Muñoz-Fontela et al., 2008; Menendez et al., 2011). IRF5 activates the transcription of pro-inflammatory cytokines (Honda and Taniguchi, 2006) and can mediate apoptosis in cancer cells (Barnes et al., 2003; Hu et al., 2005; Yanai et al., 2007; Couzinet et al., 2008; Hu and Barnes, 2009). Interestingly, IRF9 is thought to contribute to p53-mediated upregulation of IFNs in response to viruses (Muñoz-Fontela et al., 2008), but may also promote cancer cell survival by enhancing IL6 expression and STAT3 activation (Nan et al., 2018).

The Fas receptor is a p53 target and death receptor that is expressed on the surface of many different cell types and induces apoptosis upon ligand binding (Yamada et al., 2017). Examination of lpr (lymphoproliferation) and gld (generalized lymphoproliferative disease) mice with mutations in the Fas or FasL gene, respectively, revealed that Fas receptor defects cause loss of immune tolerance, an accumulation of CD4^−^CD8^−^ T-cells, and production of autoantibodies (Takahashi et al., 1994; Sobel, 1996). Together this suggests that Fas and FasL expression in T-cells mediates activation-induced cell death (Yamada et al., 2017) and that both have a critical role in T-cell development.

PKR may be regulated by p53 and contributes to p53-mediated tumor suppression under genotoxic conditions (Yoon et al., 2009), though controversial findings necessitate further investigation of the p53-PKR relationship (Rahman et al., 2009). In addition to supporting p53-mediated tumor suppression, PKR mediates inflammatory signals such as inflammasome activation and subsequent release of the inflammatory protein high mobility group box 1 (HMGB1) (Lu et al., 2012).

NK cells express NKG2D receptors that bind to ULBP1/2 ligands on the tumor cell surface. ULBP1/2 ligands are direct p53 target genes that enhance NK cell–mediated target cell recognition (Textor et al., 2011).

p53 is an important regulator of CCL2, though there is conflicting evidence pointing to both p53-dependent induction (Hacke et al., 2010) and suppression (Tang et al., 2012; Walton et al., 2016). The role of CCL2 in the tumor microenvironment is complex but much evidence points to an immunosuppressive role (Fei et al., 2021). Further investigation of the role of CCL2 and its regulation by p53 is needed to determine potential therapeutic strategies.

The cGAS/STING pathway is a key mediator of inflammation in response to cytosolic microbial and host DNA (Decout et al., 2021). Briefly, this pathway involves recognition of cytosolic DNA by cGAS, which generates the second messenger 2′,3′-cGAMP. 2′,3′-cGAMP binds to STING, which triggers the phosphorylation of IRF3 via TBK1. IRF3 then translocates to the nucleus and activates the transcription of inflammatory genes including IFNs which play a key role in tumor suppression (Du et al., 2021; Ke et al., 2022). Treatment of lung cancer cells with actinomycin D and nutlin-3a primes cells for production of type I IFN by upregulating STING in a p53-dependent manner (Krześniak et al., 2020; Pu et al., 2021), suggesting a critical role of p53 in regulating STING levels to allow for type I IFN-mediated immune responses. These types of experiments must be replicated in other cancer types to determine heterogeneity of this effect across different tumor sites.

### 2.2 p53 impacts immune cells directly

Several studies have found that p53 can alter the abundance and activation state of cells within the TME. For example, activation of wild-type p53 can reverse an immunosuppressed TME via elimination of myeloid-derived suppressor cells (MDSCs) by inducing cell death and/or reversal of their immunosuppressive capacity (Guo et al., 2017). p53 can also upregulate the NK cell ligand ULBP2 on cancer cells, which enhances NK cell anti-tumor activity (Li et al., 2011; Textor et al., 2011).

Wild-type p53 can induce TAP1 to enhance the transport of MHC class I and expression of surface MHC-peptide complexes on tumor cells. p53 also cooperates with IFN-γ to activate the MHC class I pathway. These TAP1 and IFN-γ-mediated effects are abrogated in p53-null cells (Zhu et al., 1999). Another study found that p53 upregulates MHC class I expression via upregulation of the endoplasmic reticulum aminopeptidase ERAP1 (Wang et al., 2013). The functional impact of these p53-dependent effects on cytotoxic T-cell activation and tumor regression needs to be validated *in vivo*.

p53-mediated induction of cell cycle arrest and the DNA damage response is essential for immunoglobulin generation through variability, diversity, and joining (VDJ) recombination. VDJ recombination involves the generation of double-stranded DNA breaks in B cells, thus constant monitoring of genomic integrity is crucial (Dujka et al., 2010). One study found that p53 binds to the regulatory regions of immunoglobulin genes in B cells to regulate their maturation, function, and ability to perform VDJ recombination. p53-null mice had increased splenic white pulp, higher numbers of immature B cells in their bone marrow, and B cells that were hyperresponsive to proliferative challenge. Immunoglobulin deposition was lower in tumors that developed spontaneously in p53-null mice as compared to tumors from p53+/− mice (Shick et al., 1997). Together, these findings further support the crucial role of p53 in B cell function and immunoglobulin formation.

PD-L1 has become increasingly important since the discovery and approval of ICI therapy for treatment of cancer. PD-L1 is an immune checkpoint protein that is expressed by cancer cells, macrophages, some activated T-cells and B cells, DCs and some epithelial cells. PD-L1 binds to programmed cell death protein 1 (PD-1), which is expressed on immune cells including cytotoxic CD8^+^ T-cells, and causes T-cell inactivation (Han et al., 2020). Several ICI therapies target this interaction by binding to PD-L1 or PD-1 to stimulate an anti-tumor immune response, though it must be noted that ICI therapies can cause diarrhea, colitis, hypothyroidism, hyperthyroidism, pneumonitis, and myocarditis, (Wang D. Y. et al., 2018), can increase risk of sepsis in individuals who are already at risk for this condition (Zhang et al., 2023), and occasionally cause mucositis (Jacob et al., 2021).

High expression of PD-1 or PD-L1 is a reliable predictor of favorable response to ICI across multiple cancer types. Interestingly, the relationship between p53 and PD-L1 expression varies across different cancer types. In non-small cell lung cancer, the p53-inducible miR-34 degrades PD-L1 mRNA (Cortez et al., 2016; Wang Y. et al., 2018). In melanoma cells, however, p53 can positively regulate IFN-γ-induced PD-L1 expression by boosting JAK2 expression (Yadollahi et al., 2021). Careful evaluation of PD-L1 expression after administration of p53-activating therapies should be considered before concurrent or subsequent administration of PD-L1-targeting therapy. Furthermore, the effects of p53 activation on other immune checkpoint proteins such as CTLA-4, LAG-3, and TIM-3 need to be investigated in order to identify ideal combinations with other types of ICI drugs.

Though most research has focused on the contribution of cancer cell p53 to anti-tumor immunity, some studies have investigated the role of immune cell p53. For example, there is evidence to suggest that knocking out p53 in cytotoxic T-cells leads to T-cell activation and significant enhancement of melanoma tumor control in mice (Banerjee et al., 2016). Further investigation in a wider range of cancer types could provide important guidance in the construction of chimeric antigen receptor (CAR)-T cell therapies and p53 vaccines that deliver wild-type p53 to immune cells. This type of work may also inform the administration of p53 activators that may have off-target effects on immune cells.

### 2.3 p53, the DNA damage response, and immunity

In addition to direct pharmacological activation, p53 can also be activated with DNA-damaging agents that induce apoptosis in rapidly dividing cells and/or cells with deficient DNA repair pathways. p53-mediated cell death after DNA damage can induce the immune system, a phenomenon known as immunogenic cell death (ICD) (Zhou et al., 2019). ICD can induce long-lasting protective immunity and synergize with or sensitize patients to immunotherapy (Dosset et al., 2018). Many questions remain as far as optimal time to administer immunotherapy following treatment with a DNA-damaging agent, and whether concurrent, sequential, or alternating treatment is ideal.

It is well-known that p53 target gene selection is highly context-dependent, thus the target genes that induce a p53-dependent immune response likely vary depending on the type of DNA-damaging agent that is being administered. For example, in HCT116 cells treated with chemotherapies 5-FU, irinotecan, oxaliplatin, cisplatin, or clinically relevant combinations, the soluble form of the p53 target TRAIL-R2 was downregulated in a drug-specific manner (Carlsen et al., 2021), but in breast cancer cells was upregulated in a pan-drug manner (Groysman et al., 2021). The p53 target gene TLR3, which plays a role in anti-tumor immunity, was upregulated in an irinotecan-specific manner in colorectal cancer cells (Carlsen et al., 2021). The contribution of these genes to chemotherapy-mediated immune responses remains to be investigated.

The abscopal effect describes a phenomenon in which local radiation treatment induces tumor suppression at distant sites, presumably through activation of a systemic anti-tumor immune response. One *in vivo* study demonstrated delayed growth of Lewis lung carcinoma and fibrosarcoma tumors after irradiation of a non-tumor bearing leg of mice with wild-type p53. The same experiment was repeated on p53 null mice, but normal tumor growth was observed (Camphausen et al., 2003). Another study found that irradiation of a non-tumor site induced regression of A549 tumors, but not p53-silenced A549 or p53-null H1299 tumors. This study was conducted in nude mice, which lack functional T-cells but have an intact innate immune system, pointing to the innate immune system as a major mediator of the p53-dependent abscopal effect (Tesei et al., 2021). These data support the role of p53 in anti-tumor immune responses and supports the use of radiation therapy with immunotherapy to bolster the abscopal effect (Liu et al., 2018).

There is evidence to suggest that radiation therapy can convert the tumor into an *in situ* vaccine (Formenti and Demaria, 2012). One study found that in a murine model of colorectal cancer, radiation and a CEA-targeting vaccine therapy were ineffective alone, but when combined elicited CD4^+^ and CD8^+^ T-cell responses against CEA, p53, and gp70 (Chakraborty et al., 2004). The use of radiation to upregulate tumor antigens in combination with vaccine therapies hold promise and should be investigated further.

### 2.4 p53-induced senescence and immunity

Senescence is a state of irreversible cell cycle arrest that can be induced by the p53 transcriptional program. The senescence-associated secretory phenotype (SASP) describes the highly specific secretome of senescent cells, which is composed of both pro- and anti-tumor cytokines. The role of the SASP in cancer is context-dependent. It can stimulate immune-mediated clearance of tumor cells and limit fibrosis but can also mediate chronic inflammation and stimulate the growth and survival of tumor cells (Lopes-Paciencia et al., 2019). Further investigation of SASP-mediated immune responses is needed.

p21 is a p53 target gene that plays a critical role in p53-induced cell cycle arrest (El-Deiry et al., 1993; el-Deiry et al., 1994) and is a marker of senescence along with increased levels of p16, p53, p-p53 and decreased levels of pRb (González-Gualda et al., 2021). Recent investigation has pointed to a novel role of p21 in the regulation of an early form of the SASP through Rb-dependent transcription involving SMAD and STAT. The secretome induced by this transcriptional program is known as the p21-associated secretory phenotype (PASP) and is initiated early in the stress response in parallel with cell cycle arrest. The PASP is made up of several hundred factors including CXCL14, which recruits macrophages to the tumor site. If p21 levels do not stabilize after 4 days, macrophages polarize toward the M1 phenotype and cytotoxic T-cells are recruited to eliminate the target cell. Notably, p21 overexpression recruited macrophages, T-cells, and B cells, but did not have any effect on NK cell recruitment (Sturmlechner et al., 2021). Future studies should aim to identify the extent to which this p21-dependent immunosurveillance mechanism is functional in cells with mutated p53 and if necessary, how to reactivate it.

A study by Iannello, et al. demonstrated that induction of p53 caused senescent liver cancer cells to secrete several chemokines involved in the recruitment of immune cells including CCL2, CCL4, CCL5, CCL3, CXCL1, and CXCL2. Neutralization of CCL2 prevented NK cell recruitment to the tumors and reduced tumor rejection, indicating an important role of p53-mediated CCL2 in NK cell killing of senescent tumors (Iannello et al., 2013).

## 3 Mutant p53 and the anti-cancer immune response

The TME includes cancer cells, immune cells, fibroblasts, signaling molecules, blood vessels, and extracellular matrix. The TME directly determines the immune status of the tumor. Mutant p53 can impact immune cell infiltration, cytokine secretion, and inflammatory pathways in the TME, thereby significantly impacting the anti-cancer immune response (Blagih et al., 2020a).

### 3.1 Mutant p53 and its impact on immune cell recruitment and cytokine secretion

Immune surveillance involves innate and adaptive immune responses that recruit immune cells such as CD8^+^ and CD4^+^ T-cells, NK cells, and neutrophils to clear tumor cells. Immune surveillance can be suppressed by cell types such as regulatory T-cells, MDSCs, and M2 macrophages (Swann and Smyth, 2007). Loss or alteration of p53 in cancer cells can modulate the recruitment and activation of immune cells in the tumor microenvironment and can impact cytokine secretion within the TME, resulting in the suppression or evasion of anti-tumor immune responses and the promotion of cancer progression.

Many studies show a correlation between mutant p53 and absence or reduction of cytotoxic immune cells. TCGA RNA-seq gene expression profiling revealed a decrease in the CD8^+^ T-cell marker gene (CD8A), NK cell marker genes (KLRC1 and KLRF1), T-cell cytolytic activity genes (GZMA and PRF1) in p53-mutated gastric cancers when compared to wild-type p53 gastric cancers (Jiang et al., 2018). A similar correlation between mutant p53 and reduced levels of granzyme B and perforin 1, which are mainly secreted by NK cells and cytotoxic T-cells, was observed in human head and neck squamous cell cancer based on global gene expression profiling using three multi-omics datasets (Lyu et al., 2019). Murine models further demonstrated that p53 missense mutation G242A (corresponding to human G245A) suppresses the activation of host NK cells, enabling breast cancer cells to avoid immune assault (Uddin et al., 2022).

T regs, MDSCs, and type 2 macrophages (M2) sustain pro-tumor inflammation and immunosuppression (Kerkar and Restifo, 2012). p53 dysfunction in tumor cells alters myeloid and T-cell recruitment to the tumor, promoting an immune-suppressed environment (Blagih et al., 2020b; Shi and Jiang, 2021). Higher density of myeloid cells tend to be observed in p53-mutated prostate, ovarian and breast cancers as compared to tumors with wild-type p53 (Blagih et al., 2020b). Moreover, Sallman, et al. demonstrated that mutant p53 mediates an immunosuppressive phenotype in myelodysplastic syndromes (MDS) and secondary acute myeloid leukemia (sAML). In biopsies from tumors with p53 mutations, there were less OX40+ cytotoxic T-cells and helper T-cells, decreased ICOS+ and 4-1BB+ NK cells, and expanded populations of T regs and PD-1-low MDSCs. PD-L1 expression was also significantly higher in the hematopoietic stem cells of patients with TP53 mutations (Sallman et al., 2020).

Mutant p53-associated microRNAs can also impact the immune microenvionment. Cooks, et al. discovered that colon cancer cells with gain-of-function p53 mutations secrete exosomes containing miR-1246. When these exosomes were taken up by nearby macrophages, miR-1246 reprogrammed the macrophage to a cancer-promoting state characterized by anti-inflammatory immunosuppression and increased TGF-β activity (Cooks et al., 2018).

p53 loss is associated with increased expression of the myeloid attractant cytokine CCL2 and infiltration of immunosuppressive myeloid cell populations including M2 macrophages into primary tumors (Walton et al., 2016). Another study showed that UV-induced p53 in mouse macrophages hindered LPS-induced CCL2 production (Tang et al., 2012). However, other work has shown that p53-targeting siRNA decreased levels of TNF-α-induced CCL2 transcription, therefore context specificity across different cancers should be investigated further.

The recruitment and function of immune cells in the TME are impacted by the tumor secretome, which is affected by loss or alteration of p53. For example, p53 loss in cancer cells induces secretion of WNT ligands that stimulate macrophages to produce IL-1β, promoting systemic inflammation (Wellenstein et al., 2019). Another study showed that CXCR3/CCR2-associated chemokines and M-CSF are increased in p53-deficient cancer cells. CCR2 and M-CSF then lead to the recruitment of suppressive myeloid CD11b+ cells which attenuate the CD4^+^ T helper 1 (Th1) and CD8^+^ T-cell responses in TME (Blagih et al., 2020b). Other work has shown that CXCL17, an attractant for monocytic cells, increased after p53 loss (Bezzi et al., 2018). The net effect of the mutant p53-mediated secretome on the immune status of the TME is a crucial topic of future investigation that may unveil potential targets and liquid biomarkers.

### 3.2 Mutant p53 influences inflammatory signaling pathways in cancer

Mice harboring a germline p53 mutation develop severe chronic inflammation and are highly prone to inflammation-associated colon cancer (Cooks et al., 2013), indicating a role of p53 in regulating inflammatory signaling pathways in cancer. The following sections will discuss the impact of p53 mutations on the cGAS/STING, NF-κB, and STAT3 pathways.

#### 3.2.1 The cGAS/STING pathway

As discussed above, the cGAS-STING pathway is a crucial component of the anti-tumor immune response and p53 may upregulate STING, providing important support for this tumor suppressive pathway (Krześniak et al., 2020; Pu et al., 2021). Mutant p53 interrupts the cGAS-STING pathway by interacting with TBK1, preventing formation of the STING-TBK1-IRF3 trimeric complex and blunting TBK1-dependent activation of IRF3. IRF3 is a transcriptional regulator of genes involved in type I IFN production and is critical in the cGAS/STING-mediated immune response (Ghosh et al., 2021; Ghosh et al., 2022).

#### 3.2.2 The NF-κB pathway

NF-κB is a transcription factor that senses intrinsic cell stress and regulates signaling pathways involved in inflammatory responses and tumor growth. The direct interaction between mutant p53 and NF-κB is demonstrated by genome-wide global profiling analysis, which suggest NF-κB interaction with p53 mutants (such as R273H, R248W, R248Q, and G245S) in different human cancer cell lines (Rahnamoun et al., 2017). There is a reciprocal antagonistic relationship between p53 and NF-κB, such that p53 loss results in uncontrolled NF-κB signaling and an overreaction to pro-inflammatory stimuli (Gudkov et al., 2011). Mutant p53-mediated upregulation of NF-κB signaling is observed in various human tumors. For example, mutant p53 prolongs TNF-α-induced NF-κB activation (Cooks et al., 2013) through interaction with the tumor suppressor disabled 2-interacting protein (DAB2IP) in the cytoplasm (Di Minin et al., 2014). Mutant p53 R172H can form a complex with NF-κB and activate NF-κB target gene expression in the cell lines isolated from primary pancreatic ductal adenocarcinomas of mice (Schneider et al., 2010). The interplay between mutant p53 and NF-κB reshapes cancer-promoting gene expression and cytokine secretion, driving cell transformation and cancer development. However, the role of NF-κB in the immune response is highly pleiotropic and there is evidence to support both NF-κB-mediated activation and suppression of the anti-tumor response in certain contexts (Lalle et al., 2021). Careful consideration of context-specificity should be taken before clinical translation of relevant targets.

#### 3.2.3 The TGF-β pathway

TGF-β is a pleiotropic cytokine that regulates the transcription of genes involved in myriad processes including survival, growth, proliferation, differentiation, and motility in a context-dependent manner (Elston and Inman, 2012). TGF-β signaling is highly dependent on the SMAD signal transduction pathway. Following TGF-β receptor activation, SMAD2 and SMAD3 become phosphorylated, dissociate from the receptor, and bind to SMAD4. This SMAD2/3/4 complex translocates to the nucleus to control tumor suppressor gene transcription (Elston and Inman, 2012). TGF-β impacts the TME by contributing to fibrosis, invasion, metastasis, angiogenesis, and immunosuppression (Chung et al., 2021).

Wild-type p53 and TGF-β signaling converge due to physical interaction between p53 and SMAD proteins, which induces the transcription of many tumor suppressor genes. Mutant p53, however, abrogates this effect and promotes tumorigenic TGF-β signaling that supports migration, metastasis, and p21 downregulation leading to failure of cell cycle arrest (Kalo et al., 2007; Ross and Hill, 2008; Wilkins-Port et al., 2009; Elston and Inman, 2012). These effects depend on mutant p53-mediated suppression of TGF-β receptor (TGF-βR) II expression, SMAD2 phosphorylation, SMAD2/3-SMAD4 association, and SMAD nuclear translocation (Elston and Inman, 2012). In addition to these mechanisms, oncogenic Ras and mutant p53 can work in concert with TGF-β to induce a mutant p53-p63-SMAD2/3 complex that empowers TGF-β-induced metastasis (Adorno et al., 2009) by inhibiting p63-mediated sharp-1 downregulation (Alvarez et al., 2019). Another study found that p53-mutant tumor cells secreted exosomes containing miR-21-3p and miR-769-3p that increased secretion of TGF-β in fibroblasts and induced epithelial-mesenchymal transition in tumor cells (Ju et al., 2019). Together, these studies emphasize mutant p53-mediated enhancement of pro-tumorigenic TGF-β pathways that enhance migration, metastasis, and inhibition of cell cycle arrest.

TGF-β affects CD8^+^ and CD4^+^ T-cells, B cells, and T regs in various ways to promote immunosuppression. Many of these functions are mediated by SMAD proteins. The interplay between TGF-β, mutant p53, and SMAD proteins suggest that mutant p53 impacts TGF-β-mediated immune modulation (Chung et al., 2021). Future studies should address this role of mutant p53, as it could reveal biomarkers of TGF-β-mediated immune dysfunction and relevant therapeutic targets.

Wild-type p53 interactions with TGF-β can also promote worse patient outcomes. In certain contexts, TGF-β-mediated p53 upregulation leads to enhanced DNA damage repair and resistance to radiotherapy (Petroni et al., 2022). TGF-β can mediate radiation-induced lung fibrosis, however the involvement of p53 has not been investigated (Gans et al., 2021). One study found that pulsed low dose rate radiation induced lower levels of TGF-β and less tissue atrophy when compared to conventional radiation, pointing to a potential solution to this dose-limiting side effect (Meyer et al., 2017). As p53 is highly differentially expressed in tissues exposed to radiation therapy, the role of p53 in TGF-β-mediated lung injury after radiation should be investigated to identify the potential of p53-targeting therapies to further prevent radiation-induced fibrosis.

#### 3.2.4 The STAT3 pathway

STAT3 is a transcription factor that mediates cancer inflammation and promotes cancer cell survival via regulation of various cytokines and growth factors. STAT3 can bind to the p53 promoter to inhibit its transcriptional function (Niu et al., 2005) and p53 loss in turn leads to IL-6-mediated STAT3 phosphorylation and activation (Wormann et al., 2016). Further, STAT3 and NF-κB act synergistically to activate the transcription of FAT10, a gene that can counteract p53 function (Choi et al., 2014). As its role in the immune response is more well-defined than NF-κB, targeting p53 LOF-mediated STAT3 activation to suppress both STAT3 and NF-κB pro-cancer signaling may be a viable approach to inducing an anti-tumor immune response.

### 3.3 p53 as a tumor antigen

p53 is a tumor antigen that can differentiate cancer cells from normal cells. This was first recognized when researchers identified anti-p53 antibodies in mice with chemically-induced tumors (DeLeo et al., 1979). After this initial discovery, numerous studies have contributed evidence supporting the role of mutant p53 as a tumor antigen and the therapeutic potential of targeting mutant p53 with adoptive cell therapies (McCarty et al., 1998). Further, point mutations on p53 in cancer cells generate neoantigens (Chasov et al., 2021) that can improve response to immunotherapy. For example, Deniger, et al. found that p53 hotspot mutations (c.659A>G; p.Y220C and c.733G>A; p.G245S) expressed by two different patients’ ovarian tumors were individually immunogenic (Deniger et al., 2018). In patients with lung adenocarcinoma, tumors with p53 mutations had higher PD-L1 expression and higher levels of tumor-infiltrating cytotoxic T-cells as compared to tumors with wild-type p53 (Dong et al., 2017; Biton et al., 2018; Deniger et al., 2018).

Survivin is a member of the inhibitor of apoptosis (IAP) family and is negatively regulated by wild-type p53 (Hoffman et al., 2002). When p53 is mutated, survivin levels increase, making it mutant p53-mediated tumor antigen (Tang et al., 2021). Survivin protein presented by DCs can induce cytotoxic lymphocytes *in vitro* (Schmitz et al., 2000) and prime cytotoxic lymphocytes in an *in vivo* murine melanoma model (Hofmann et al., 2009), suggesting its potential as an antigen target for immunotherapy.

The relative contribution of mutant p53-dependent neoantigen generation and immune suppression to the overall state of the TME likely varies across cancer type and subtype. Identifying personalized clinical approaches to targeting mutant p53 to stimulate the immune response requires careful investigation.

## 4 Clinical translation of p53 activation to induce an immune response

Due to the tumor suppressive role of p53 in apoptosis, cell cycle arrest, and activation of the immune system, reactivating wild-type p53 or restoring wild-type function to mutant p53 has great clinical potential for cancer treatment (Zhang et al., 2022). While numerous clinical trials have tested the safety and efficacy of p53-activating therapies, few have evaluated immune responses after treatment or combined p53-activating therapies with immunotherapy (Table 2). The following section discusses clinical progress so far. The results from these clinical trials have been variable, with achievement of an immune response in some patients but with little evidence of lasting tumor regression.

APG-115 (alrizomadlin) is an orally active small molecule inhibitor that activates p53 by interfering with the MDM2-p53 protein-protein interaction. Preclinical data demonstrated that APG-115 activates wild-type p53 in immune cells in the TME to promote anti-tumor immunity regardless of p53 status in the tumor cells (Fang et al., 2019). Ongoing clinical trials are testing the combination of APG-115 with the anti-PD-1 antibodies toripalimab (NCT04785196) or pembrolizumab (NCT03611868). Results from NCT03611868 indicate that APG-115 + pembrolizumab is well tolerated and demonstrated preliminary antitumor activity in multiple tumor types (McKean et al., 2022). Another trial is evaluating the combination of pembrolizumab with the p53 activating compound PRIMA-1Met (APR-246, eprenetapopt), which had an acceptable safety profile and showed clinical activity in patients with solid tumors (NCT04383938) (Park et al., 2022; Dumbrava et al., 2021). ALRN-6924 is a p53-reactivating peptide that demonstrated enhancement of the anti-cancer immune response even in the absence of immunotherapy *in vivo.* There are two completed (NCT02264613, NCT02909972) and two active (NCT03654716, NCT03725436) clinical trials evaluating this peptide, though none note an intention to evaluate the immune response to treatment (Zhou et al., 2021).

Some p53-activating therapies involve vaccines that deliver wild-type p53 to cells such as APCs. For example, p53MVA is a genetically engineered vaccinia Ankara viral vector that expresses a wild-type p53 transgene. This vaccine delivers full-length p53 to APCs to generate T-cell responses against p53 epitopes and stimulate p53-specific IFN-γ-secreting CD8^+^ T-cells that proliferate and exhibit cytolytic function against p53-overexpressing tumor cells *in vitro*. Single-agent p53MVA was administered to advanced, refractory colon and pancreatic cancer in a phase I clinical trial (NCT01191684) and was well-tolerated and elevated p53-specific CD8^+^ T-cell responses (Chung et al., 2013; Hardwick et al., 2014). Combining this therapy with the TLR9 agonists CpG deoxynuceotides (CPG-ODN) or CTLA-4 blockade resulted in tumor rejection *in vivo* but this strategy remains to be translated (Blagih et al., 2020a). Other approaches involve ALVAC-p53, which is a recombinant canarypoxvirus vaccine encoding wild-type human p53. ALVAC-p53 induced p53-specific immune responses in colorectal cancer patients (van der Burg et al., 2002). Two clinical trials investigating a p53-synthetic long peptide (SLP-p53) vaccine in combination with IFN-α2b (NCT01639885) or cyclophosphamide (NCT00844506) have been completed, but results have not been posted.

## 5 Future directions

Multiple clinical trials have investigated the combination of p53-activating therapies with immunotherapy (Table 2). Results have been variable, with achievement of an immune response in some patients but with little evidence of lasting tumor regression. The multifaceted contribution of p53 to the anti-cancer immune response provides numerous therapeutic targets in cancers with mutated p53 and an immunosuppressed TME, however these targets likely vary depending on the type of cancer and p53 mutation. For example, p53 can upregulate PD-L1 to enhance efficacy of anti-PD-L1 and anti-PD-1 ICI, but this effect varies across cancer type (Wang Y. et al., 2018; Yadollahi et al., 2021). Genetic alteration of p53 disrupts cancer cell secretion of pro-inflammatory cytokines (Bezzi et al., 2018; Wellenstein et al., 2019; Blagih et al., 2020b) and enhancement of antigen presentation (Zhu et al., 1999; Wang et al., 2013), but mutant p53 can also enhance the generation of neoantigens (Chasov et al., 2021) that supports response to ICI. The relative contribution of these effects likely varies across different types of cancer and p53 mutations. Further investigation of context-specificity across cancer type, mutation type, and cell type is needed for optimal clinical translation of p53 restoration to induce an immune response in immune-suppressed TMEs.

There is evidence to suggest that treatment of tumors with DNA damaging agents can sensitize cancer cells to immunotherapy in certain contexts by upregulating PD-L1 or inducing ICD (Dosset et al., 2018). The contribution of p53 to these mechanisms remains to be clarified and much work remains to identify optimal dosing and treatment timing when combining DNA damaging agents and immunotherapy to induce a maximal immune response.

Other types of immunotherapies besides ICI and p53 vaccines such as adoptive cell therapy, monoclonal antibodies, oncolytic viruses, and immune system modulators have demonstrated efficacy in various cancer types but have not been tested in combination with p53-reactivating therapies. It would be relevant to test their efficacy in combination with p53-reactiving compounds in cancers with mutant p53. Emerging technologies should also be considered, such as bispecific antibodies that can efficiently target specific p53 mutants for T cell-mediated death (Hsiue et al., 2021).

A recent study identified an MDM2/MDM4/MDMX-independent link between p53 loss and hyperprogression after pembrolizumab treatment in a murine model of mismatch repair-deficient colorectal cancer (Sahin et al., 2021). As up to 30% of patients experience hyperprogression after immunotherapy treatment, elucidating the mechanisms behind this phenomenon is highly clinically relevant. It is important to consider p53-dependent prevention of hyperprogression in these types of studies (Okan Cakir et al., 2019).

In conclusion, p53 may modulate the anti-cancer immune response via direct activation of immunomodulatory genes, interaction with the cGAS/STING, NF-κB, TGF-β, and STAT3 signaling pathways, direct and indirect effects on immune cells, modulation of PD-L1 and immunomodulatory cytokine levels, and regulation of the senescence-associated secretory phenotype. Both wild-type and mutant p53 are tumor antigens that can be therapeutically targeted and certain p53 mutants may predict enhanced response to immune checkpoint inhibition. It is likely that the relative contribution of each of these p53-dependent mechanisms to the anti-cancer immune response varies across different cancer types with various genetic backgrounds and tumor microenvironments. Further investigation with careful consideration of experimental conditions is needed to identify optimal personalized treatment strategies for cancer patients.

## Figures and Tables

**Table:** 

**CAR-T**	Chimeric antigen receptor
**cGAS**	Cyclic GMP–AMP synthase
**DC**	Dendritic cell
**DR5**	Death receptor 5
**Gld**	Generalized lymphoproliferative disease
**HMGB1**	High-mobility group box 1
**IAP**	Inhibitor of apoptosis
**ICD**	Immunogenic cell death
**ICI**	Immune checkpoint inhibition
**IFN**	Interferon
**IRF**	Interferon regulatory factor
**Lpr**	Lymphoproliferation
**LUAD**	Lung adenocarcinoma
**MDSC**	Myeloid-derived suppressor cells
**MHC**	Major histocompatibility complex
**NK**	Natural killer
**NKT**	Natural killer T
**PASP**	P21-associated secretory phenotype
**PD-1**	Programmed cell death protein 1
**PD-L1**	Programmed death-ligand 1
**SASP**	Senescence-associated secretory phenotype
**SLP**	Synthetic long peptide
**STING**	Stimulator of interferon genes
**TGF-βR**	TGF-β receptor
**Th1**	T helper 1
**TLR**	Toll like receptor
**TME**	Tumor microenvironment
**TRAIL**	Tumor necrosis factor-related apoptosis-inducing ligand
**TRAIL-R2**	Tumor necrosis-factor related apoptosis-inducing ligand receptor 2
**VDJ**	Variability, diversity, and joining
